# Intra-Abdominal Heterotopic Cardiac Xenotransplantation: Pearls and Pitfalls

**DOI:** 10.3389/fcvm.2019.00095

**Published:** 2019-07-25

**Authors:** Laura DiChiacchio, Avneesh K. Singh, Joshua L. Chan, Nicole M. Shockcor, Tianshu Zhang, Billeta G. Lewis, David Ayares, Philip Corcoran, Keith A. Horvath, Muhammad M. Mohiuddin

**Affiliations:** ^1^Department of Surgery, University of Maryland Medical Center, Baltimore, MD, United States; ^2^National Heart, Lung, Blood Institute, National Institute of Health, Bethesda, MD, United States; ^3^Revivicor, Inc., Blacksburg, VA, United States

**Keywords:** xenotransplant, xenograft, cardiac, pig, baboon, abdominal, heterotopic, complications

## Abstract

Heterotopic cardiac xenotransplantation in the intra-abdominal position has been studied extensively in a pig-to-baboon model to define the optimal donor genetics and immunosuppressive regimen to prevent xenograft rejection. Extensive investigation using this model is a necessary stepping stone toward the development of a life-supporting animal model, with the ultimate goal of demonstrating suitability for clinical cardiac xenotransplantation trials. Aspects of surgical technique, pre- and post-operative care, graft monitoring, and minimization of infectious risk have all required refinement and optimization of heterotopic cardiac xenotransplantation over time. This review details non-immunologic obstacles relevant to this model described by our group and in the literature, as well as strategies that have been developed to address these specific challenges.

## Introduction

Heterotopic cardiac xenotransplantation model is widely used to study the immunology of xenograft rejection and to test various immunosuppressive regimens. This model has several advantages as it facilitates immunologic monitoring through the period of graft rejection at reduced cost and complexity compared to orthotopic models. Since the native heart remains in place, rejection of heterotopic xenograft does not result in primary hemodynamic compromise. This model has additionally allowed for the successful demonstration of multiple immunosuppression regimens that have resulted in long-term xenograft survival (1–4). However, due to its non-physiologic nature, it does not offer information as to whether the organ can sustain life.

Multiple challenges affect the successful transplantation of a heterotopic cardiac xenograft. Immunologic complications have been a major hurdle to long-term graft and recipient survival, as both bacterial and fungal infections due to non-specific immune suppression can be commonly seen (5). Viral pathogens, such as cytomegalovirus (CMV) herpes simplex virus (HSV) and simian T-cell leukemia virus (STLV) are common in baboons, although this has been lessened with the implementation of specific-pathogen-free baboons, early weaning, and serologic screening (6, 7). All donor pigs are weaned early and demonstrate negative serology for porcine CMV prior to transplant. Thromboembolic complications are also linked to this model, due to *de novo* cross-species incompatibilities as well as the use of therapeutic immunosuppressive agents, such as anti-CD154 ligand antibody (8–11). Systemic coagulopathies similar to disseminated intravascular coagulation (DIC) are also shown to be associated with delayed rejection of pig xenograft in the pig-to-baboon species combination (12). Understanding these common complications as well as the tools with which to overcome them is essential to the success of this model.

This paper describes our group's experience and the lessons learned with the pig-to-baboon intra-abdominal heterotopic cardiac xenotransplant model, with a cohort of 88 transplants to date, and provides a systemic review of available literature. Results from our group are from both the National Institutes of Health and University of Maryland Medical Center and the experiments detailed in this review were approved by the respective Institutional Animal Care and Use Committees.

## Technical Considerations

The intra-abdominal heterotopic model is a two-anastomosis system utilizing arterial supply from the infrarenal aorta of the recipient baboon to perfuse the coronaries of the donor pig heart with drainage of the xenograft through the donor pulmonary artery remnant anastomosed to the abdominal inferior vena cava of the recipient. During procurement of the donor heart, arrhythmias and prolonged warm ischemia time must be avoided. Pretreatment with amiodarone (15 mg) is our standard for dysrhythmia prophylaxis. Heparinization is achieved with 500 IU/kg 3 min prior to cross-clamp. Diastolic arrest is achieved with 20–30 cc/kg of cold crystalloid cardioplegia, followed by cold static ischemia. We generally use Custodial HTK, but equivalent commercial preservation solutions, such as Belzer UW are acceptable.

During implantation, care must be taken to protect bowel and bladder during exposure of the abdominal aorta at the level of the iliac bifurcation. Additionally, geometric considerations during implantation of the graft are key, as kinking of either donor aorta or pulmonary artery can lead to inflow or outflow obstruction that may result in stasis, thrombus formation, and graft failure. The pulmonary artery to inferior vena cava anastomosis is particularly susceptible to thrombosis from angulation at the time of abdominal closure. As in an orthotopic position, de-airing of the heart and establishment of normal sinus rhythm are integral to long-term graft survival, and electrocardioversion is occasionally required to establish such a rhythm as described in the arrhythmias in donor heart section below. Adams et al. described early experience with avulsion of the aortic suture line immediately upon post-operative movement of the baboon into an upright position. The authors report that particular care with replacement of bowel loops into anatomic position prior to closure prevented any further disruption of the graft secondary to shifting of intra-abdominal contents (13).

## Post-Operative Management

A number of supportive measures are required to maintain optimal function of a heterotopic xenograft that would not be utilized in an orthotopic model due to its non-load bearing, relatively low-flow state. Continuous intravenous heparin is required to keep the activated clotting time to twice its baseline level to minimize clot formation. This is particularly imperative with longer surviving xenografts as formation of left ventricular thrombi are more frequently observed. In addition, continuous infusion of heparin has been demonstrated to have a mitigating effect on the development of consumptive coagulopathy, and has been utilized in other models, including the kidney xenograft model (14).

Cefazolin is routinely utilized for bacterial prophylaxis for 1 week following placement of a central venous catheter (Bard Medical, Covington, GA). This line is maintained until the xenograft is explanted to allow for delivery of medications over extended periods of time (e.g., continuous heparin, 2-h infusion of MMF, etc.), access for routine blood draws, or administration of therapies during emergent circumstances. The central venous catheter traverses through a jacket and tether system (Lomir Biomedical, Inc.) that protects it from inadvertent damage by the baboon and allows for access by the research team from outside the baboon cage. The long-term tethering system permits relative mobility of the animal while still providing durable and reliable intravenous access for the research team (15). Over time, multiple improvements have been implemented, including improvement in the durability of the jackets, strengthening the critical attachment of tether to the jacket, and redesigning the swivel system to reduce friction. We have additionally identified that providing a period of time for the animal to acclimatize to both jacket and tether prior to the start of the experiment improved the lifespan of the system.

Implanted telemetry (DSI, St. Paul, MN) as described elsewhere (16) is utilized for early detection of infection, in the form of central temperature monitoring, and graft rejection, through measurement of left ventricular pressure and continuous electrocardiography. This has been utilized for monitoring of continuous EKG in this model since its inception (13). Interval monitoring with blood work, manual palpation of the graft, as well as graft echocardiography is undertaken. Close monitoring allows for prompt treatment of developing infection, thrombus formation and in rare cases graft rejection. In cases where telemetry, laboratory, physical exam, and ultrasonography suggest rejection, there is a role for open biopsy in this model. This is done only when absolutely necessary per institutional animal care committee restrictions and is generally performed at the time of potential explant.

## Weight Matching for the Intra-Abdominal Position

**Figure 1:**
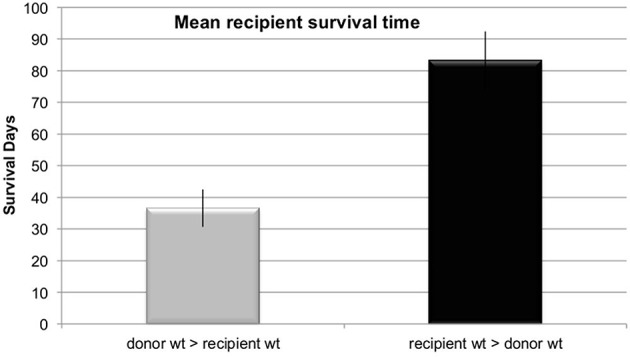
Weight matching donors and recipients. Mean recipient survival, in days, stratified by donor weight greater than or less than recipient weight, respectively.

The disparity between the donor and the recipient size plays an important role in subsequent development of complications. A heart from a smaller pig (3–4 weeks old ~5–6 kg in weight) transplanted in a larger baboon (>10 kg) allows enough room for the heart to function without compression and fibrillation at abdominal closure. On the other hand, a larger heart in a smaller baboon appears to compress the graft, restricting its systolic function, abdominal compartment syndrome with hypotension of the recipient and induces arrhythmias leading to loss of the xenograft if not recognized and treated promptly. The relatively small size of available recipient baboons and our preference to use smaller baboons to save on costly immunosuppressive drugs limits the size of potential donor pig hearts. In our group's experience, grafts from larger donor pigs become compressed by the recipient's abdominal wall during closure, resulting in elevated abdominal compartment pressures. Figure 1 compares survival of grafts based on donor and recipient weights. The disparity between the donor and the recipient size plays an important role in development of subsequent complications. As a general rule, a donor pig of ~80% of the recipient baboon weight allows enough room for the heart to function without compression at abdominal closure. Xenografts greater than this size ratio frequently resulted in decreased systolic function, abdominal compartment syndrome, and lethal cardiac arrhythmias.

Size matching in the heterotopic model has always been a challenge, even prompting early groups to utilize cervical implantation as an alternative (13, 17). While case reports of successful transplantation with a donor-recipient weight mismatch of more than 20% have been published, other series demonstrate that a donor:recipient weight ratio of <0.66 results in increasing morbidity and mortality in the recipient (18). Another consideration is the health and intrinsic cardiac function of transgenic pigs at various sizes; this is a potential confounder of these outcomes. Nevertheless, we have found that with further experience in size matching, and technology for non-invasive graft monitoring, abdominal heterotopic placement of the cardiac xenograft has become easily accessible and manageable.

## Bleeding Complications

Hemorrhagic complications in our experience are attributed to several causes. Consumptive coagulopathy is a known sequela of xenograft placement and generally occurs within 4 weeks of transplantation. In our early experience, this occurred in ~10% of our cases and is discussed in more detail in *Coagulation Dysfunction*, below. The need for continuous heparin infusion further increases the risk of bleeding in the xenotransplant setting.

**Figure 2:**
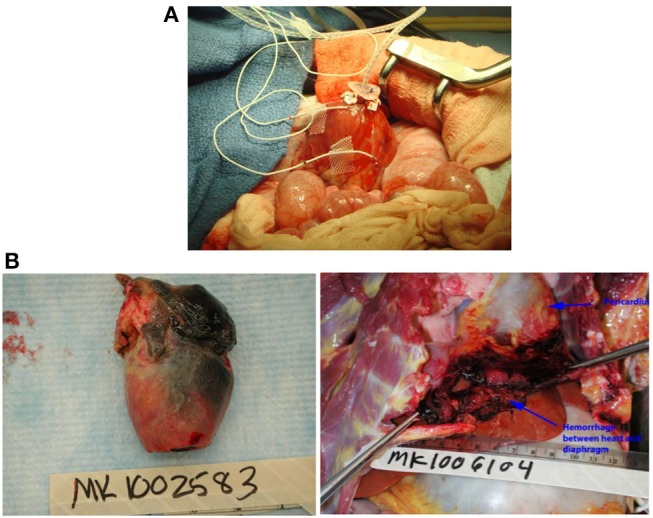
**(A)** Placement of left ventricular probe. A pursestring of pledgetted Prolene sutures is placed to secure the LV probe at the apex. **(B)** Complications of graft ischemia. Cardiac infarction **(left)** and rupture with intra-abdominal hemorrhage **(right)**.

In some initial experiments, the site of the LV telemetry probe bled; this was prevented in later transplants by securing the probe tightly with pledgetted Prolene sutures (Figure 2A). In two cases a region of the left ventricle failed to reperfuse, became infarcted, and ruptured within 2–3 weeks, leading to recipient fatality (Figure 2B). In one case bleeding was the result of a ruptured mesenteric vessel that had adhered to the transplanted graft. There was no technical error identified in this case on necropsy. In our earlier cases when anti-inflammatory drugs were not used, mesenteric and bowel adhesions around the transplanted heart were a common occurrence as described below, and in one very active baboon it led to bleeding from these adhesions.

## Thromboembolic Complications

Use of costimulation blockade of the CD40/CD154 pathway for induction and maintenance immunosuppressive therapy has led to the recent profound prolongation of graft survival. Early experience utilizing anti-CD154 antibody was successful in immunomodulation but led to an unacceptable incidence of complications from thromboembolic events (11). Phase II trials using anti-CD154 antibody to treat lupus and kidney transplant recipients were forced to end early due to adverse events from thromboembolic complications (19, 20). These effects are thought to be secondary to antibody-binding of CD154 expressed on activated platelets, increasing platelet aggregation and thrombus formation (10, 11). Early use of anti-CD154 in our group utilized ketorolac in conjunction to decrease the degree of platelet aggregation has had encouraging results. We have now transitioned to the use of anti-CD40 antibody in place of anti-CD154 for costimulation blockade. This has significantly reduced the rate of thromboembolic complications while maintaining effective immunosuppression and favorable xenograft survival (21). In addition, anticoagulation with systemic unfractionated heparin is used routinely in our recipients to target an activated clotting time 2 × the baseline to prevent thrombus formation.

## Intestinal Adhesion and Related Complications

**Figure 3:**
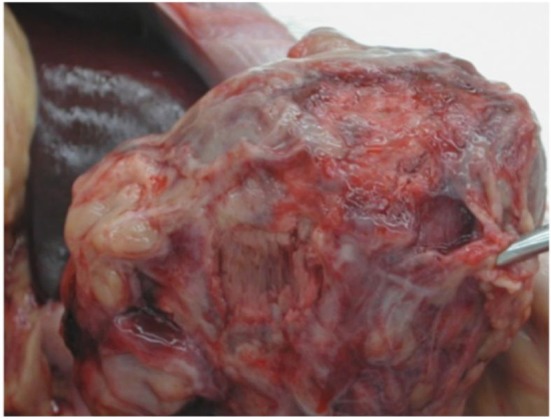
Formation of adhesions. Dense, diffuse intestinal adhesions apparent surrounding graft.

In addition to the bleeding associated with the single case of adhesed mesenteric vessel rupture, intestinal adhesions are a risk specific to this model. While adhesions are a risk associated with any abdominal operation, the inflammation associated with xenograft placement confers a particularly high risk of significant adhesion formation. As shown in Figure 3, adhesions of omentum and small bowel to the xenograft were observed in almost all transplants; in a few cases, these adhesions were sufficiently severe to contribute to recipient fatality. One intestinal obstruction led to aspiration and sudden death; another very active, otherwise healthy recipient expired as a result of intestinal perforation. Theoretically, an adhesion barrier could be utilized to minimize the risks associated with intra-abdominal adhesion formation; personal experience with adhesion barriers have shown no difference in adhesion formation, likely due to the presence of exceptionally high levels of inflammation in this model. We have seen markedly reduced adhesions lately as we have routinely started using anti-inflammatory drugs in our regimen, including IL-6R and TNFα inhibitors.

## Arrhythmias in the Donor Heart

Atrial and ventricular fibrillation, ventricular tachycardia and bundle branch block were witnessed in conditions when larger hearts were used in relatively small baboons. Pig hearts, and genetically-modified (GM) pig hearts in particular, are more prone to fibrillation than human or non-human primate hearts. Subtle differences in ion channel expression in the GM pig heart are hypothesized to account for this increased myocardial irritability (22). We utilize a cocktail of mannitol, lidocaine, and sodium bicarbonate during reperfusion to reduce this risk. When fibrillation does present, it occurs immediately after reperfusion, during the closure of the abdominal cavity, or within 12 h post-operatively. Arrhythmias are treated with an amiodarone bolus (15 mg) followed by initiation of a continuous amiodarone infusion (15 mg/24 h) and electrical cardioversion as necessary. Because electrocardioversion results in the long-term telemetry device becoming inoperable due to the spike in current, cardioversion was not used in a number of late cases of fibrillation, resulting in loss of graft function without rejection in all of these cases. In some cases, when detected early, the implant was removed, the heart cardioverted into sinus rhythm, the device re-implanted and the abdomen closed without further incident.

## Coagulation Dysfunction

**Figure 4:**
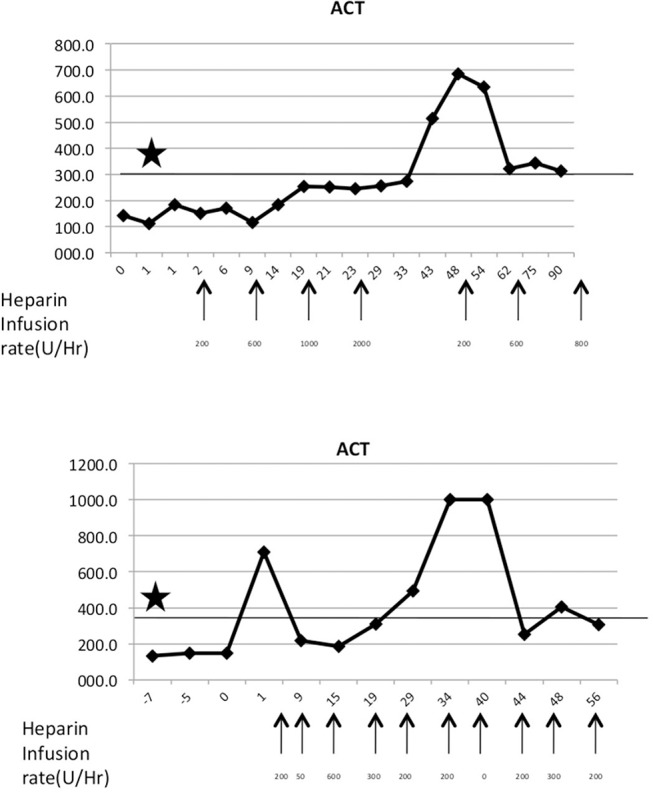
Maintenance of hypercoagulable state. **(Top)** Baboon with a hypercoagulable state with heparin dosages indicated by arrows. Asterisks denote target ACT levels. **(Bottom)** A normal baboon with episodic ACT hikes.

Two types of disorders were observed related to dysregulated coagulation. The consumptive coagulopathy typically seen in this model has been described by others (23–25); however, platelet consumption and uncontrollable bleeding were only observed in two baboons with functioning grafts. One baboon demonstrated hypercoagulability without thrombocytopenia or hemorrhage, which manifested as thrombosis in jugular vessels repeatedly leading to occlusion of two IV catheters. A very large dose of heparin infusion (1,000 U/h compared to 50 U/h usual dose for an 8 kg baboon) was required to prevent coagulation and recurrent line thrombosis. Two baboons experienced a sharp decrease in platelet count and hematocrit 21 and 28 days after transplant, respectively, and responded well to transfusion of allogeneic packed red blood cells. However, more than three transfusions also precipitated severe coagulopathy leading to recipient mortality. A tight control of ACT and platelet count in two baboons is demonstrated in Figure 4.

We observed a drop in hematocrit (HCT) to 15% in four recipients who also developed bruising over their abdomen. The platelet numbers in these baboons also dropped to 50–70,000/mm^3^. Initially small amounts of packed red cell transfusion (60–70 ml) transiently improved the condition but was not able to sustain the affect. Later we were able to transfuse large quantities of blood (20 ml/kg packed RBC's) that greatly helped in restoring the HCT to normal. Over a period of time, platelet numbers also improved in these baboons. In three cases where multiple (>2; 8–20 ml packed RBC/kg) blood transfusions were deemed clinically necessary the recipient succumbed in association with uncontrolled consumptive coagulopathy.

## Infectious Complications

**Figure 5:**
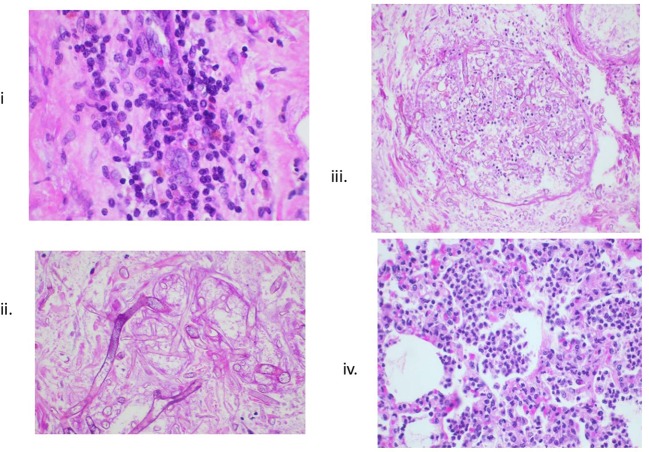
Histologic evidence of infections. (i) Lymphocytic infiltrates in transplanted heart; (ii, iii) Zygomycosis; and (iv) bronchopneumonia.

Infectious complications are of paramount significance in the immunosuppressed recipient. Routine use of prophylactics (Cefazolin for the first week following transplant and ganciclovir throughout the duration of immunosuppression) is mandatory in our model. Additionally, long-term telemetry device placement allows for continuous body temperature recording such that any febrile episodes followed by blood culture were promptly evaluated and treated. In fact, the benefit of the implanted telemetry device, primarily designed for direct pressure monitoring of the graft, was primarily through the early detection of central temperature spikes. This allowed us to overcome the majority of potential infections complications efficiently with the use of broad-spectrum antibiotics in any recipient exhibiting a fever >39°C. One case of fungal infection (Zygomycosis) did occur that was ultimately diagnosed by identification of fungus on histological analysis (Figure 5). No clinically significant Cytomegalovirus infections occurred. While attention to the long-term tunneled central line is important, we do not routinely exchange these lines, nor have we experienced line-related infections in this model.

One early series, prior to the use of implantable telemetry and continuous temperature monitoring, demonstrated 23 episodes of infection in a series of 16 baboons; 14 of these episodes resolved with treatment, although unfortunately 8 resulted in death of the recipient (5). In this series the mean time from transplant to infection was 44 days. All CMV infections (6) were with baboon CMV and were associated with low levels of ganciclovir. No episodes of fungal infection were observed in this series.

We have found with the use of early intervention based on results from continuous non-invasive monitoring that clinically significant infections are rare, and controlled effectively with bacterial, viral, and fungal prophylaxis, as well as prompt treatment of fever with broad-spectrum coverage (26, 27).

## Sudden Death

Two baboons suffered sudden death without any prior sign and symptoms. Necropsy excluded acute hemorrhage, pulmonary embolus, pneumonia, and intra-abdominal catastrophe, but did not yield any conclusive evidence regarding the cause of death. Ventricular fibrillation is a potential etiology that could not be excluded on post-mortem exam.

## Conclusions

The heterotopic transplant model has served as a useful tool to study the immunological barriers to xenotransplantation. This model has also allowed testing and refinement of several immunosuppressive and immunomodulatory agents and approaches, and demonstrated the clear benefits associated with various genetic modifications to donor pigs. While a number of complications remain in this model, the immunologic success has formed the foundation for experiments in the orthotopic model. The successful survival of the intra-abdominal heterotopic cardiac xenograft requires vigilance by the scientific team; so, too, will success in the orthotopic model.

We recently reported the longest survival of 945 days in a heterotopic xenotransplantation model utilizing either GTKO pigs that also express human CD46 and thrombomodulin (GTKO.hCD46.TBM) (21, 28). That result was achieved using an immunosuppressive regimen that is a combination of regimens reported earlier, based on co-stimulation blockade with anti-CD40, MMF and anti-CD20 antibody. Herein, we describe challenges in the use of this model that are unrelated to immunosuppression. We have little direct evidence that the immunosuppression, which appeared to have been largely sufficient to surpass the anti-non-Gal and other immune barriers, was lethal to the recipient, since viral and bacterial infections were not common. Recipient morbidity and mortality were in many cases attributable to the heterotopic placement of the heart and possibly to the inflammatory rejection process. Initially, a number of recipients in this experiment died before complete rejection of the heart xenograft due to some avoidable complications. Important lessons were learned from these experiments and these complications were prevented from recurring.

Some of the early hurdles were related to the tether, animal jacketing and jugular intravenous catheters. These were overcome following several modifications. We faced the problem of consumptive coagulopathy and thromboembolism in a few baboons but mostly these complications occurred within the first post-transplantation month. Some of the late complications included sudden death, uncommon infections and abdominal bleeding due to rupture of the transplanted heart or mesenteric vessels adherent to the xenograft. While the animals did undergo multiple procedures with multiple cardiac stressors, no evidence of native cardiomyopathy or depressed myocardial function in the native heart was observed in our experiments.

Typical consumptive coagulopathy reported by many was not common in our experiment. We did notice signs similar to consumptive coagulopathy in two baboons. These symptoms were overcome in one case with a series of packed red cell infusions to restore normal coagulation profile. However, three other cases resulted in demise of these baboons. Based on this, we recommend that persistent or recurrent anemia in this model should be treated by graft removal or euthanasia. The coagulopathy observed was limited to a sharp decrease in platelet numbers and elevation of activated clotting time of blood. This early drop in platelet numbers is likely due to deposition in the graft. When the graft survives this insult, the numbers normalize on their own. This is inconsistent with an immune-mediated process, such as heparin-induced antibody mediated thrombocytopenia, which we have not seen in our experiments.

In one case thrombosis of the intravenous catheter and subsequent occlusion of a major vessel also hampered the success of this experiment. Some baboons required higher doses of heparin infusion (up to 2,000 U/h) to achieve target ACT levels of 2 × baseline. Thrombotic microangiopathy related to anti CD154 has been described (29) but in our study we did not come across this complication in the absence of graft dysfunction. Perhaps routine administration of ketorolac immediately before the administration of this antibody prevented this complication as previously reported (7).

Intestinal adhesions to the heterotopic heart caused recipient mortality in two recipients, due to bowel obstruction with aspiration, intestinal rupture, and mesenteric vessel erosion. These complications led to recipient baboon's mortality before the transplanted heart was rejected. In additional cases, bowel obstruction due to intestinal adhesions was relieved surgically when identified early by abdominal X-ray, and we recommend use of X-ray imaging early for any baboon with difficulty tolerating a diet, lethargy, or signs of discomfort or systemic illness without decreased graft function.

The use of telemetry implant helped accurate measurement of graft function and facilitated early detection of graft dysfunction (ventricular fibrillation, decreased contractility in association with immunologic injury). The heart rate and ventricular contractility based on left ventricular peak systolic and end diastolic pressure accurately and non-invasively measured graft function. The temperature probe was very useful in detecting the early onset of infection and these infections were treated at an early stage. Therefore, this complication was not a significant factor in our experiments, in contrast to experiences reported by others (5). Continuous treatment with intravenous Ganciclovir also was effective to prevent baboon CMV infection.

In conclusion, long-term xenograft survival has been achieved in a large animal heterotopic model where the loss of recipients to model-associated complications often used to occurs before the loss of graft function. While most of these complications are avoided in our recent experiments, we conclude that, based on encouraging results with current GM pig lines and immunosuppressive regimens, the orthotopic model may offer new challenges but a more productive format as the basis for developing a clinically applicable regimen. With continued vigilance and experience with the intra-abdominal heterotopic model, with new GM pigs and novel target specific immunosuppressive agents, even further advancement toward a successful orthotopic xenotransplant model is within reach.

## Data Availability

All datasets generated for this study are included in the manuscript and/or the supplementary files.

## Ethics Statement

Results from our group are from both the National Institutes of Health and University of Maryland Medical Center and the experiments detailed in this review were approved by the respective Institutional Animal Care and Use Committees.

## Author's Note

MM, AS, BL, and LD are part of the Cardiac Xenotransplantation Program at the University of Maryland.

## Author Contributions

LD participated in the performance of the research and wrote the paper. AS participated in the design and performance of the research and participated in writing and editing the paper. JC participated in the performance of the research, reviewed and edited the paper. NS and TZ participated in the performance of the research and writing the paper. BL participated in the design and performance of the research. DA, PC, and KH participated in the design of the research and in the final approval of the paper. MM designed and performed the research, participated in writing the paper, and provided final approval of the paper.

### Conflict of Interest Statement

DA is the CEO and President of Revivicor, Inc. The authors declare that funding was received from United Therapeutics Inc for this project. The manuscript was also approved for submission by United Therapeutics. The funder had no role in study design, data collection, data analysis, and preparation of manuscript. However, the main material (donor pigs) were provided by them and based on our agreement with them, they had to approve the manuscript for publication. All authors had part of their salary driven from this funding but had no other potential conflict of interest.
